# Thunder-fire moxibustion for cervical spondylotic radiculopathy: Study protocol for a randomized controlled trial

**DOI:** 10.1186/s13063-019-4012-1

**Published:** 2020-02-06

**Authors:** Yunxuan Huang, Jiabi Zhang, Buhui Xiong, Ruina Huang, Wenjing Zhao, Mengxue Zhou, Qi Chen, Danghan Xu, Xinghua Chen

**Affiliations:** 1 The First Affiliated Hospital of Guangzhou University of Chinese Medicine, No.12 Ji Chang Road, Baiyun District, Guangzhou, 510405 China; 2 Guangzhou University of Chinese Medicine, No.12 Ji Chang Road, Baiyun District, Guangzhou, 510405 China; 3 The Eighth Affiliated Hospital of Sun Yat-sen University, No. 3025 Shennan Middle Road, Futian District, Shenzhen, 518033 China; 4 Shenzhen Pingle Orthopaedic Hospital, No.252 Hangzi Section, Pingshan Avenue, Pingshan District, Shenzhen, 518118 China; 5 Kyoto Prefectural University of Medicine, Kajii-cho, Kawaramachi-Hirokoji, Kamigyo-ku, Kyoto, 602-8566 Japan

**Keywords:** Cervical spondylotic radiculopathy, Thunder-fire moxibustion, Ibuprofen, RCT

## Abstract

**Background:**

Thunder-fire moxibustion originated in China and contains traditional Chinese medicine. It can produce strong firepower, infrared thermal radiation, and medicinal effects when burning on the acupoints. Thunder-fire moxibustion is commonly used in patients with neck pain, but its efficacy has rarely been systematically demonstrated. We designed a randomized trial of thunder-fire moxibustion on cervical spondylotic radiculopathy (CSR) to investigate whether it is more effective than ibuprofen sustained-release capsules.

**Methods:**

One hundred patients will be recruited and randomly divided into thunder-fire moxibustion and ibuprofen groups. The intervention consists of ten treatments and will last for 2 weeks. The Yasuhisa Tanaka 20 Score Scale is used as the primary outcome measure. It contains a combination of the self-conscious symptom in patients, objective clinical evaluation from doctors, and social evaluation (the ability to work and live). The objective and comprehensive evaluation of CSR patients before and after treatment is particularly needed. The Short-Form McGill Pain Questionnaire-2 (SF-MPQ-2), Neck Disability Index score scale (NDI), and the Quality of Life Assessment (SF-36) are applied as secondary outcome measures. The assessment will take place at the baseline and the first and second weekends of treatment. If an adverse event (AEs) occurs, it will be reported.

**Discussion:**

The aim of this trial is to determine whether thunder-fire moxibustion is more effective than ibuprofen in the treatment of patients with CSR.

**Trial registration:**

Chinese Clinical Trial Registry (http://www.chictr.org.cn), ChiCTR1800018820. Registered on 11 October 2018.

## Background

Cervical spondylotic radiculopathy (CSR) is characterized by the dysfunction of a cervical spinal nerve, the roots of the nerve, or both [1, 2]. Patients with cervical spondylotic radiculopathy suffer deeply from numbness and pain of the arm and neck. According to the Global Burden of Disease Study (2013) [3], among 301 chronic and acute injuries and illnesses in 188 countries, neck pain was one of the top ten causes of disability of years. Furthermore, neck activities are restricted. CSR, as a common type of cervical spondylosis (CS), accounts for about 60 to 70% [4].

Currently, the approved CSR treatment strategies contain surgical and non-surgical treatment, including traction, drugs, functional exercise, and physical therapy, etc. [5, 6].

In practice, analgesics are standard primary treatments for CSR unless there is evidence of spinal cord disease or apparent inability to move. Nonsteroidal anti-inflammatory drugs (NSAIDs) could relieve pain as first-line agents in acute settings [7]. The efficacy of ibuprofen in the treatment of cervical nerve root pain has also been reported [8], but it can only relieve pain and has no effect on the other symptoms of CSR.

In the treatment of CS neck pain, complementary therapies such as acupuncture, moxibustion, and massage have been widely accepted [9]. Thunder-fire moxibustion, which contains refined moxa as well as agarwood, frankincense, costus root, and other traditional Chinese medicines, is often used in China to warm the meridian to relieve pain [10]. However, the effect of thunder-fire moxibustion on CSR remains uncertain because of poor study design and small sample sizes in previous clinical trials [10–13].

In this study, the target is to investigate and compare the efficacy of thunder-fire moxibustion with oral application of ibuprofen sustained-release capsules in patients with CSR suffering from pain, numbness, and dysfunction.

## Methods

### Trial design

We have designed a single-center randomized controlled trial (RCT) to compare thunder-fire moxibustion with ibuprofen in CSR patients. Participants will receive ten treatments within 2 weeks.

**Fig. 1:**
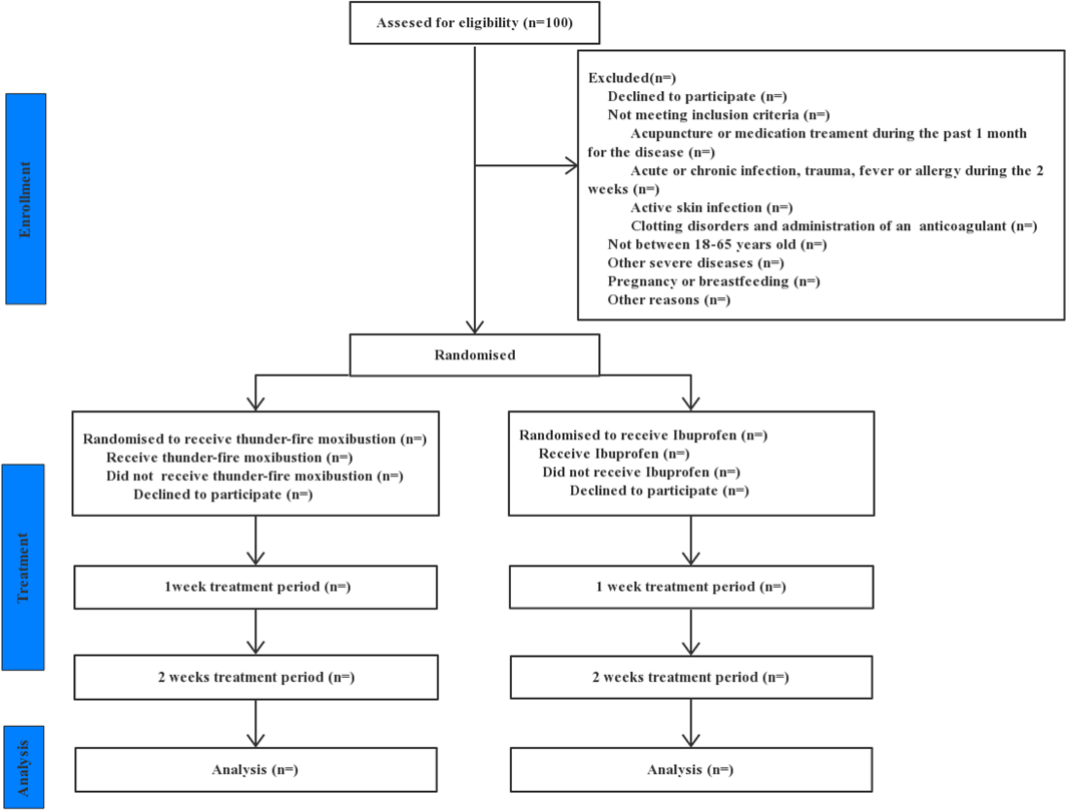
Trial flow chart

One hundred patients with CSR will be recruited and randomly assigned to either the thunder-fire moxibustion group or an ibuprofen group by a 1:1 ratio (Fig. 1). Our study is based on the rule of common clinical trials (Declaration of Helsinki). The SPIRIT checklist is given in Additional file 1

.

### Inclusion criteria

Participants will be enrolled with the following criteria in this study: (1) men or women aged from 18 to 65 years, (2) the main signs and symptoms are numbness and pain along spinal nerve roots, (3) intervertebral foramen extrusion and/or brachial plexus pull tests is/are positive,(4) the clinical manifestations and imaging are in compliance with the clinical syndromes, and (5) participants who can understand the scales, i.e., those used to measure trial outcomes.

### Exclusion criteria

Subjects will be excluded if they meet one of the following criteria: (1) tennis elbow, tendonitis of biceps brachii, periarthritis of the shoulder, acute spinal cord injury, or syndrome of cubital tunnel, carpal tunnel, thoracic outlet; (2) symptoms of cervical vertigo, acute spinal cord inflammation, and abnormal changes on transcranial Doppler (TCD); (3) acupuncture treatment or medication for the disease during the past 1 month; (4)acute or chronic infection, trauma, fever or allergy during the previous 2 weeks; (5) any other situations, which includes taking anticoagulant, clotting disorders, active skin infection; (6) severe primary disease or infectious diseases including kidney, heart or liver disease, hematopoietic system, endocrine system disease, or pregnancy or breastfeeding; (7) a severe psychological disorder or psychiatric condition associated with dementia and severe neurosis and inability to communicate or take care of oneself; (8) patients who are afraid of moxibustion.

### Recruitment

We will recruit participants by advertising on bulletin boards, located at the Department of Orthopedics, the Department of Acupuncture and Characteristic Chinese Medicine, and the Department of Rehabilitation Nursing Center at the First Affiliated Hospital of Guangzhou University of Chinese Medicine. Recruitment staff working in these departments will be in charge of the recruitment and registration of the participants who meet the inclusion criteria. The details about the participants will be maintained by the Data Monitoring Committee (DMC) and will never be revealed to any other individual or organization irrelevant to this study.

### Randomization and allocation concealment

A randomized block design is used for the study. Patients with CSR will be randomly assigned to the group of thunder-fire moxibustion or ibuprofen in a 1:1 ratio. The random list will be generated by an independent statistician by a block size of 6 using SAS 9.1 software. The random numbers list generated will be concealed using opaque and sealed envelopes with an independent custodian.

### Blinding

This is an open-label study. However, researchers consisting of outcome measurers and statisticians in the trial should be blinded to reduce the impact of subjective impressions.

### Interventions

**Fig. 2:**
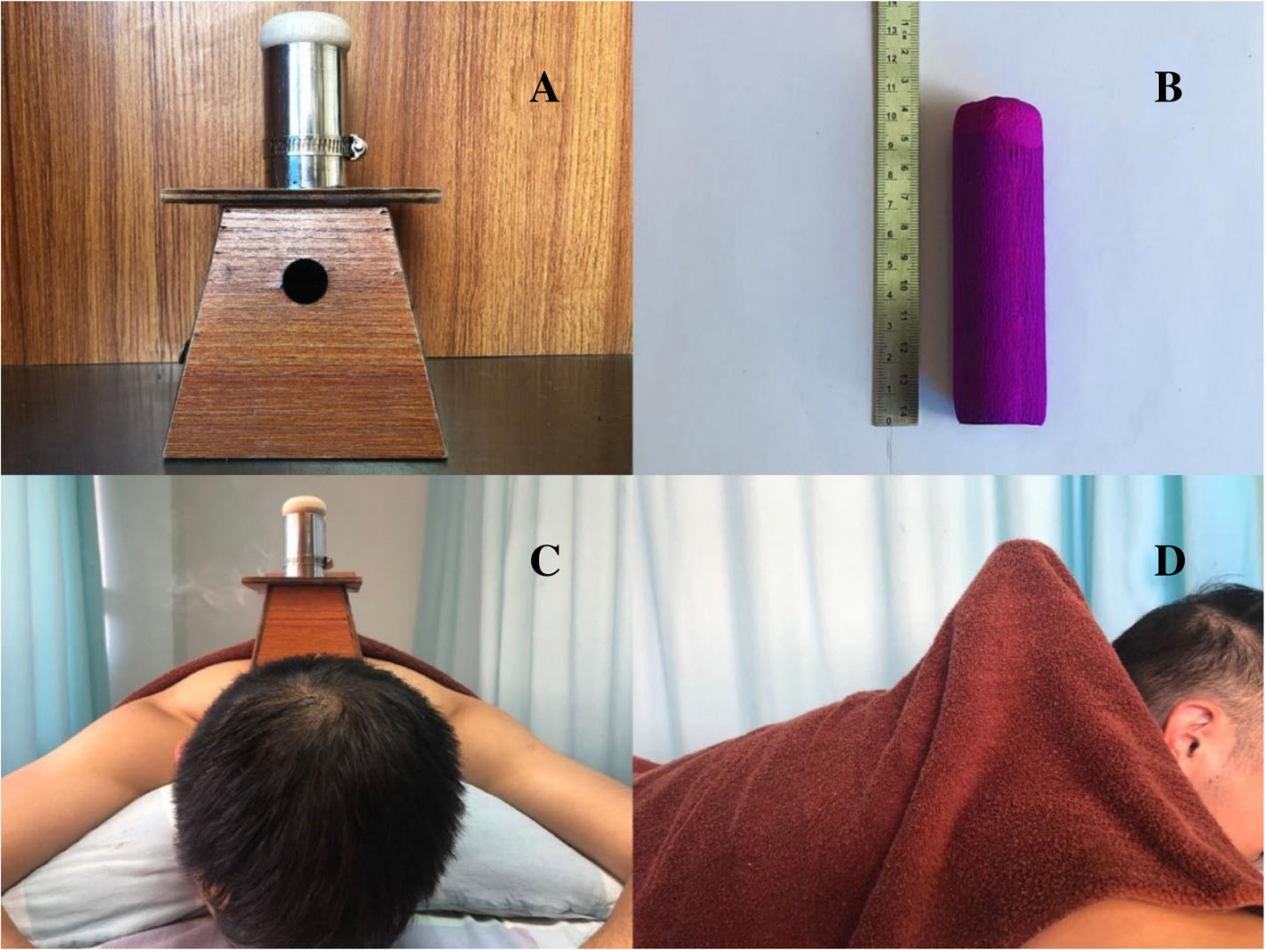
Thunder-fire moxibustion. Picture **A** is the 1-hole moxibustion box. Picture **B** is a moxa-cigar. Picture **C** shows that the moxibustion box is placed on the treatment site. Picture **D** shows that the moxibustion box and the treatment area are covered with a thick treatment towel

**Fig. 3:**
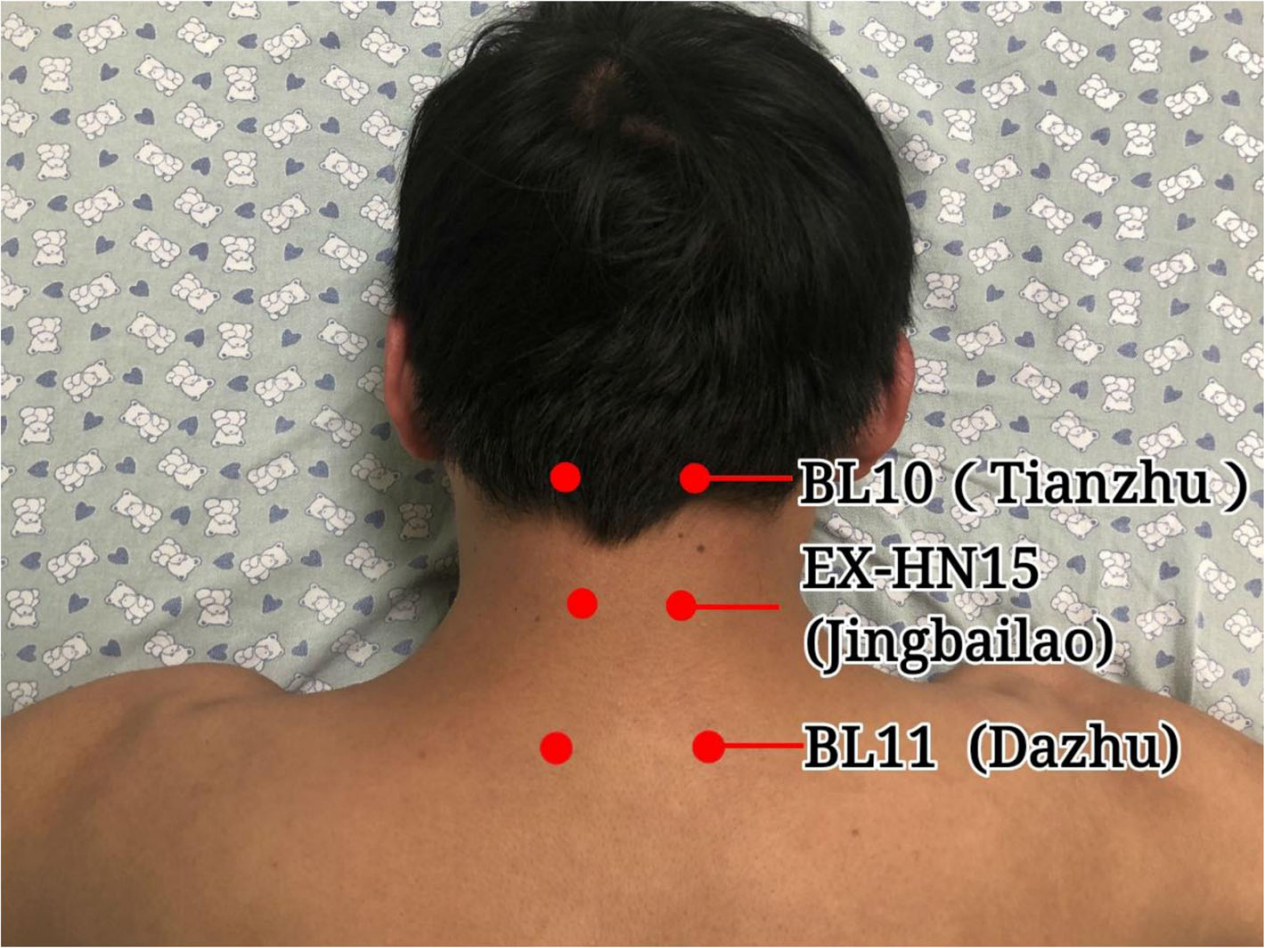
Location of acupoints

Moxibustion staff members who have more than 1-year-experience in thunder-fire moxibustion will be called for this trial and retrained for standard operation before the trial. The moxa-cigars for the moxibustion are produced by the Traditional Medicine Research Institute of Zhao’s thunder-fire moxibustion. Each moxa-cigar is 10 × 3 cm and weighs 25 g. The treatment site is the local neck, using acupoint selections BL10 (Tianzhu), BL11 (Dazhu), and EX-HN15 (Jingbailao). The subject will be in a relaxed prone position. One moxa-cigar is placed in a 1-hole moxibustion box, and the tops of the moxa-cigars are lit. The moxibustion box is then placed on the treatment site, with the fire head 2–3 cm from the skin. Moxibustion is performed on the local neck area for a treatment of 30 min. The moxibustion box and the treatment area are covered with a thick treatment towel to maintain temperature and to control smoke (Figs. 2 and 3). Treatments will be given 5 days per week for 2 weeks. The treatment using ibuprofen from SK&F (0.3 g per tablet) will also last 2 weeks, with two tablets per day taken orally.

### Outcome measurements

**Fig. 4:**
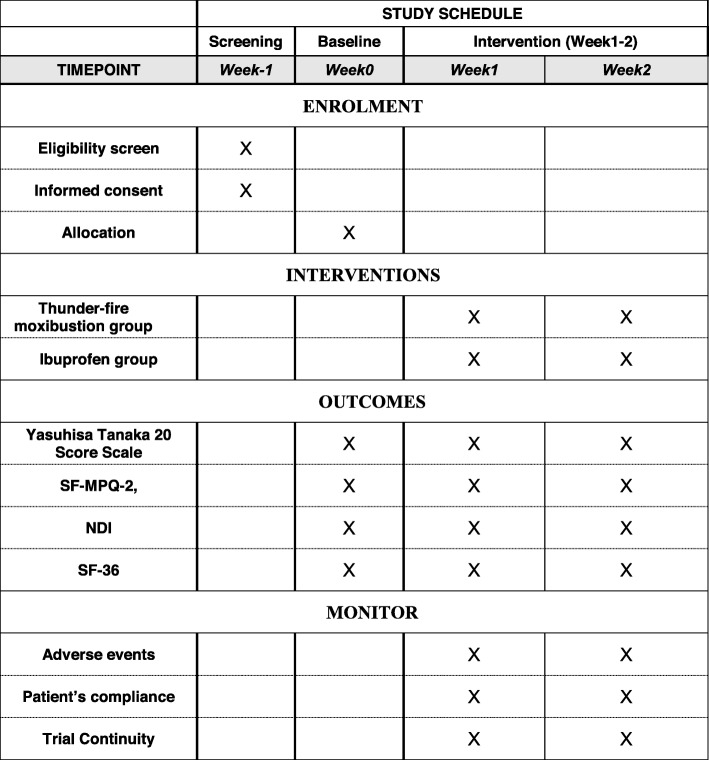
Study schedule. Week 1: the 5th treatment during the first week. Week 2: the 10th treatment during the second week. All outcomes take place at baseline, the first weekend, and the second weekend during treatment

All the outcome measurements will take place at the baseline (before treatment) and the first and second weekend during treatment (Fig. 4).

#### Primary outcome measurement

##### Yasuhisa Tanaka 20 Score Scale

The Yasuhisa Tanaka 20 Score Scale [14–16] has proven its efficiency to assess cervical spondylotic radiculopathy. It consists of nine items, which includes the severity of neck pain, stupefaction, and the pain from upper limbs or fingers. Signs include intervertebral orifice extrusion test, sensation, muscle strength, tendon reflex, and function of the hand. Working and living ability is also included. Low scores indicate worse pain and disability.

#### Secondary outcome measurement

##### Short-Form McGill Pain Questionnaire-2 (SF-MPQ-2) [17]

There are four parts in this questionnaire: (1) continuous pain such as cramping ache, aching pain, tender ache, gnawing ache, and heavy ache; (2) intermittent soreness, for instance, shooting hurt, sharp hurt, splitting hurt, stabbing hurt, and electric-shock hurt; (3) neuropathic pain including hot-burning ache, cold-freezing ache, “pins and needles” or tingling, ache caused by light touch, itching, and numbness; (4) emotional overtired, unwholesome, scared, and excruciating scale. Four different scores were added to get the final scores. A higher score represents more severe pain.

##### Neck Disability Index Score Scale (NDI) [18, 19]

Neck-specific functional disability will be measured by the original ten-item Neck Disability Index (NDI). The NDI covers ten dimensions including entertainment, sleep, driving, headache, reading, personal care, work, lifting, concentration, and pain intensity. One dimension will be assessed on each item measured by a 6-point scale from 0 (no disability) to 5 (complete disability). The scores of each item will be added and multiplied by 2 to get the final total score (out of 100).

##### Quality of Life Assessment (SF-36) [20]

The 36-Item Short-Form Health Survey (SF-36) is a concise health measurement scale developed by the Boston Institute of Health. It has eight dimensions and 36 clauses, including the dimensions physical role limitation (RP), physical functioning (PF), general health (GH), bodily pain (BP), vitality (V), emotional role limitation (RE), mental health (MH), and social functioning (SF). Questions and answers will be transformed into a point scale ranging from 0 to 100. High marks mean severe damage due to cervical spondylotic radiculopathy.

### Sample-size calculation

This study aims to estimate the exact effect of thunder-fire moxibustion compared to ibuprofen. The sample size has been estimated based on the results of a previous study that had shown an extract of acupuncture and moxibustion eases CSR [10]. The Yasuhisa Tanaka 20 Score Scale will be used as the primary outcome measure to assess the analgesic effect of thunder-fire moxibustion in this trial. Results from a previous study showed that the mean changes in the Yasuhisa Tanaka 20 Score Scale were 8.27 ± 2.48 (acupuncture and moxibustion group) and 6.77 ± 2.58 (control group) [10]. SPSS14 software was used to calculate the sample size. The confidence of the trial sample size was 90% and the significance level was 0.05. Results showed that clinically significant differences would be detected using a minimum sample size of 46 individuals in each group. The maximum permissible drop-out rate was 10% and 100 subjects (50 per group) were recruited.

### Statistical analysis

To eliminate artificial error, two statisticians will be involved to independently run statistical analysis via SPSS software (version 24.0) and R statistical package (version 3.5.0).

If the continuous variables can meet a normal distribution or T distribution, the data between the two groups will be compared by Student’s *t* test. Otherwise, we will use the Mann–Whitney test or Wilcoxon test. For categorical data, the Fisher’s exact or the Chi-square test will be adopted. It is statistically significant when the *p* value is under 0.05. We will select the intention-to-treat principle to perform this statistical analysis. Thus, multiple imputations will be used to address the missing data. We will use two different methods: intention-to-treat and per-protocol, for sensitivity analysis. Moreover, we will establish a multiple regression model to control the covariates. We can calculate the independent effect of each variable on the outcome via this model, which will provide data for our further trials.

### Safety and adverse events

The group of thunder-fire moxibustion may encounter adverse events including xerostomia, constipation, skin burn, bleeding tendency, dizziness, and fainting [21–23]. If the above situation occurs, the thunder-fire moxibustion treatment should be stopped immediately, and the participants should drink warm water and have a rest. Adverse events caused by oral ibuprofen mainly include tinnitus, dizziness, drowsiness, skin rash, nausea, vomiting, abdominal distension, itching, dyspepsia, and blurred vision. Once these adverse reactions occur, participants must stop the ibuprofen and select symptomatic treatment if necessary. These AEs will be subcategorized by severity: mild, moderate, and severe adverse events (mild adverse events = adverse events are transient and tolerable; moderate adverse events = adverse events will cause discomfort and interfere with the subject’s normal life; severe adverse events = serious impact on the participants’ physical health and even lead to the risk of life). The record form will be filled in if adverse events occur during the treatment period including the time, duration, performance, measures to be taken, and the outcome.

### Data management and monitoring

A case report form (CRF) will be used in data collection. Data information on demographics and assessment after each treatment of every participant will be recorded completely by the data monitoring committee. The cause of patient drop-out should be clarified in the CRF for all shedding cases. At the end of the study, the investigator will submit the case report form to the data management committee for all patients enrolled in the trial. Continuity of the trial will be assessed if more than 25% of the patients discontinue intervention due to moderate or severe adverse events.

The data monitoring committee is independently chaired by the Statistics Teaching and Research Office of Guangzhou University of Chinese Medicine and claims to have no conflict of interest. The South China Research Center for Acupuncture and Moxibustion will act as an independent committee to monitor the progress and provide advice if necessary. The Ethics Committee of the First Affiliated Hospital of Guangzhou University of Chinese Medicine will take part in endpoint adjudication. All staff members will be included in the author’s contribution.

The Project Management Group will meet every week to review trial conduct. The Trial Steering Group will meet every month, and the independent Data Monitoring and Ethics Committee will meet every 6 months to review conduct throughout the trial period.

## Discussion

The pathogenesis factors of CSR include cervical degeneration, trauma, strain, cervical dysplasia, inflammation, wind, cold and wet environment, etc. Modern medicine holds that CSR originates from the secondary inflammatory injury caused by nerve root stimulation and hyperplasia due to cervical disc herniation or joint hypertrophy, resulting in arm and shoulder numbness and pain [1]. Modern medicine mostly adopts drug treatment, rehabilitation treatment, and surgical treatment. Nevertheless, traditional Chinese medicine has a proven remarkable effect on the treatment of CSR with acupuncture, massage, and moxibustion.

Since ancient times, moxibustion therapy has been widely used in the prevention and treatment of a variety of systemic diseases, including digestion, exercise, respiration, cardiovascular, and urinary systems, etc. Furthermore, in clinical practice, moxibustion treatment of cervical spondylosis has achieved a certain effect. However, according to the literature, moxibustion is usually accompanied by other therapies, such as acupuncture and massage, for the treatment of cervical spondylosis. Design of these combined treatments may be due to the weaker effect of general moxibustion. Therefore, we choose thunder-fire moxibustion as our intervention, for which the penetration is stronger than the general moxibustion.

After the burning of thunder-fire moxibustion, energy, thermal infrared radiation, medicine chemical factor, and physics factor are produced, so as to adjust human body's enginery to treat disease.. Using heat radiation, thunder-fire moxibustion improves blood circulation by the heat penetrating through the tissue [9], demonstrated by a strong effect on blood stasis and swelling as well as pain relief [24]. Compared with ordinary moxa sticks, the advantages of thunder-fire moxibustion are larger in coverage, safer, simpler operation, have higher target-point positioning accuracy, are easier to control, have lower scald risk, and reduced smog moxibustion. Moreover, thunder-fire moxibustion can alleviate other symptoms of CSR such as numbness and dysfunction.

In the previous four trials [10–13], none of them used thunder-fire moxibustion alone as the study group, so that they cannot clearly demonstrate the effectiveness of thunder-fire moxibustion. Herein, we separate the thunder-fire moxibustion or ibuprofen comparison for the sake of evaluating the practical effect of thunder-fire moxibustion. Four trials [10–13] all randomly selected local lesion such as BL10 (Tianzhu), BL11 (Dazhu), EXHN15 (Jingbailao), GB20 (Fengchi), GV14 (Dazhui), GV15 (Yamen), GV16 (Fengfu), and ashi points (an acupuncture point with no specific name or definite location; the site of which is determined by tenderness or other pathological responses, also known as the ouch point). As a matter of fact, four trials were not in conformity with the acupoints selection principle of thunder-fire moxibustion. According to special moxibustion box tactics, we chose BL10 (Tianzhu), BL11 (Dazhu), and extraordinary point EX-HN15 (Jingbailao) in this study.

There are limitations to this study. One limitation is the blinding. Blinding cannot be conducted in this trial, so an open-label study is adopted, which may lead to performance bias. However, outcome measurers and statisticians will be blinded to try our best to adjust bias. Secondly, the participants will only be recruited at the First Affiliated Hospital of Guangzhou University of Chinese Medicine, and then the result of this trial may be suitable for Chinese people only.

The trial duration is short (a 2-week treatment), because we only focus on the short-term effect of thunder-fire moxibustion. Moreover, ibuprofen is not typically suitable for long-term use [25].

### Trial Status

This protocol is version 2.0. 2019-03-17. The participants will be recruited from March 1, 2019 to June 1, 2020. But no one will be enrolled until this paper is submitted.

## Supplementary information

**Additional file 1.** SPIRIT 2013 Checklist: Recommended items to address in a clinical trial protocol and related documents.

## Data Availability

The datasets analyzed during the current study are available from the corresponding author on reasonable request.

## Abbreviations

AEs: Adverse events
BP: Bodily pain
CRF: Case report form
CSR: Cervical spondylotic radiculopathy
GH: General health
MH: Mental health
NDI: Neck Disability Index
NSAIDs: Nonsteroidal anti-inflammatory drugs
PF: Physical functioning
RCT: Randomized controlled trial
RE: Emotional role limitation
RP: Physical role limitation
SF: Social functioning
SF-36: Quality of Life Assessment
SF-MPQ-2: Short Form McGill Pain Questionnaire-2
TCD: Transcranial Doppler
V: Vitality
